# Targeting 4-1BB (CD137) to enhance CD8 T cell responses with poxviruses and viral antigens

**DOI:** 10.3389/fimmu.2012.00332

**Published:** 2012-11-08

**Authors:** Yuan Zhao, Vikas Tahiliani, Shahram Salek-Ardakani, Michael Croft

**Affiliations:** Division of Immune Regulation, La Jolla Institute for Allergy and ImmunologyLa Jolla, CA, USA

**Keywords:** vaccinia virus, poxvirus, 4-1BB, CD8 T cells, memory, vaccination

## Abstract

Attenuated vaccinia virus (VACV) vectors are considered prime vaccine candidates for use in immunotherapy of infectious disease. In spite of this, recent data show that the level of attenuation may hamper the efficient generation of protective CD8 T cells. This suggests that additional adjuvant-like activities may need to be combined with attenuated VACV for optimal vaccination. Stimulatory reagents to the TNFR family molecule 4-1BB (CD137) may represent such an adjuvant for vaccination. Previous murine studies have found that 4-1BB can participate in optimal priming of effector and memory CD8 T cells in response to several virus infections, and concordantly direct stimulation of 4-1BB with agonist reagents effectively boosts the CD8 T cell response against those viruses. In contrast, we recently reported that 4-1BB plays no role in the response to a virulent strain of VACV, questioning whether agonists of 4-1BB will be useful adjuvants for vaccination with VACV vectors. Here we show that agonist anti-4-1BB strongly enhanced the primary viral-specific effector CD8 T cell response during infection with live virulent VACV and attenuated VACV, and during immunization with VACV peptides given in IFA. However, accumulation of memory CD8 T cells was enhanced only following infection with virulent VACV or with peptide vaccination, but not with attenuated VACV, correlating in part with more transient expression of 4-1BB on CD8 T cells with attenuated virus. Our data therefore suggest that 4-1BB may be a promising candidate for targeting as an adjuvant for short-term enhancement of CD8 T cell responses with VACV vaccine strategies, but additional receptors may need to be engaged with 4-1BB to allow long-term CD8 T cell immunity with attenuated VACV vectors.

## INTRODUCTION

A challenge for the development of vaccines and vectors for immunotherapy of infectious disease is to allow safe delivery of T cell epitopes that will induce efficient short or long-term cell-mediated immune responses. In particular, being able to promote CD8 T cell populations that can eliminate cells infected with viruses or intracellular pathogens is an attractive goal that is being recognized as a desirable component of any vaccine. This has led to viral vectors or subunit vaccines being considered for diseases such as HIV, hepatitis, and malaria. While many researchers might acknowledge that live vaccines or viral vectors are the best way to induce cell-mediated immunity and CD8 T cell responses, there is concern about the safety of live vaccines and in particular about adverse side effects that might result with viruses or viral vectors that replicate strongly and spread within the host. In this regard, the study of vaccinia viruses (VACV) is of relevance as they are considered to be prime candidates for use in immunotherapy. However, the VACV strains used during the eradication of smallpox induced severe adverse effects in immunocompromised patients, leading to the development of attenuated strains for clinical use. Although attenuated vaccines/vectors, that replicate poorly or do not replicate at all, solve the latter issue, recent data are questioning whether these will induce strong, optimal, and/or long-lasting T cell responses, particularly in the CD8 compartment (Morgan et al., 1988; Cleghorn et al., 2007; Karem et al., 2007; Peters et al., 2007; Thongcharoen et al., 2007; Ferrier-Rembert et al., 2008; Midgley et al., 2008). The rationale here is that the magnitude of the T cell response is often proportionately related to the amount of antigenic epitopes that are available and presented over time (Wherry et al., 2002; Liu et al., 2007, 2010; Blair et al., 2011; Salek-Ardakani et al., 2011a). Thus, poor viral replication will translate into poor induction of T cell immunity.

The question is then whether a non-replicating or attenuated viral vector, or isolated antigens, can be used together with an adjuvant mechanism that allows effective and optimal induction of CD8 T cell responses while still maintaining the safety desirable in such a vaccine. Various data published over the past 10 years has suggested that targeting stimulatory/pro-inflammatory members of the TNFR superfamily might provide this adjuvant-like activity (Watts, 2005; Salek-Ardakani and Croft, 2010; Goulding et al., 2011). For example, we showed that exogenous stimulation of the OX40 receptor (CD134), with an agonist reagent, could effectively boost CD8 T cell priming with an attenuated VACV virus, to the extent that the induced populations engendered protection against a normally lethal VACV infection that did not rely on other protective mechanisms such as antibody (Salek-Ardakani et al., 2011a,b). The rationale for targeting OX40 was that a highly virulent strain of VACV resulted in engagement of the endogenous OX40 receptor that is expressed on activated CD8 T cells, and this led to the generation of greatly elevated frequencies of primary effector and memory CD8 T cell populations compared to those induced with attenuated VACV variants (Salek-Ardakani et al., 2008, 2011a; Goulding et al., 2011). 4-1BB (CD137) is another TNFR molecule that can be induced on activated CD8 T cells, as well as CD4 T cells, NK cells, NKT cells, and some dendritic cells (Croft, 2009; Croft et al., 2012). Endogenous 4-1BB/4-1BBL interactions have been shown to participate in priming of virus-specific CD8 T cells during infection with influenza virus (Bertram et al., 2002, 2004), LCMV (Tan et al., 1999, 2000) and HSV (Seo et al., 2003), and correspondingly targeting 4-1BB with an agonist antibody, or incorporation of 4-1BBL into a vaccine vector, allowed enhanced CD8 responses to these viruses (Halstead et al., 2002; Kim et al., 2005; Zhang et al., 2007; Moraes et al., 2011; Vezys et al., 2011). Altogether this implies that knowledge of the endogenous use of a stimulatory receptor might be generally applicable for determining what molecule might be amenable for targeting to enhance vaccine efficacy with attenuated virus vectors or with isolated viral antigens.

In contrast to the positive endogenous activities reported above, we recently found no role for 4-1BB or 4-1BBL in generating primary effector or memory CD8 T cells elicited by the virulent Western Reserve strain of VACV (VACV-WR; Zhao and Croft, 2012). This then presented an interesting scenario to test whether targeting a receptor that was not normally active during an anti-viral response could still be efficacious in enhancing CD8 T cell immunity to that virus. Here, we assessed the effects of an agonist antibody to 4-1BB in promoting CD8 T cell responses to VACV variants and VACV epitopes.

## MATERIALS AND METHODS

### MICE

Eight-week-old female C57BL/6 mice were purchased from the Jackson Laboratory (Bar Harbor, ME). Average starting weight of these mice was 21.45 ± 0.93 g (*n* = 42). The studies reported here conform to the animal Welfare Act and the NIH guidelines for the care and use of animals in biomedical research. All experiments were conducted following the guidelines of the La Jolla Institute for Allergy and Immunology’s Institutional Animal Care and Use Committee.

### VIRUSES

The VACV Western Reserve and Lister (VACV-Lister) strains were purchased from the American Type Culture Collection (Manassas, VA), grown in HeLa cells, and titered on VeroE6 cells.

### IMMUNIZATION PROTOCOLS

Mice were infected i.p. with 2 × 10^4^ or 2 × 10^5^ PFU of VACV, or were immunized s.c. at the base of the tail with 2 μg or 10 μg/mouse of various CD8 T cell peptide epitopes emulsified in IFA together with a hepatitis B virus core 128–140 (TPPAYRPPNAPIL) epitope. Mice were also injected with 25 or 100 or 150 μg agonist anti-4-1BB (clone 3H3) or rat IgG (Chemicon) as control on the days stated in the figure legends.

### VACV INTRANASAL CHALLENGE

Mice were anesthetized by inhalation of isoflurane and inoculated by the intranasal route with 3.5 × 10^6^ of VACV-WR. Mice were weighed daily for 2 weeks following challenge and were euthanized when they lost more than 25% of their initial body weight and this was loss was maintained for greater than 24 h. Body weight was calculated as percentage of the mean weight for each group on the day of challenge.

### PEPTIDES AND TETRAMERS

Vaccinia virus peptide epitopes used in this study were predicted and synthesized as described previously (Tscharke et al., 2005; Moutaftsi et al., 2006); B8R (20-27; TSYKFESV), B2R (54-62; YSQVNKRYI), A23R (297-305; IGMFNLTFI). N2L (60-68; FLMMNKDEL), B16R (275-283; ISVANKIYM), MHC/peptide tetramers for the VACV-WR epitope B8R (20-27; TSYKFESV)/H-2Kb, which were conjugated to allophycocyanin, were obtained from the National Institutes of Health Tetramer Core facility (Emory University, Atlanta, GA).

### IMMUNOFLUORESCENCE LABELING

Tetramer-positive cells were identified after gating on CD8 T cells with anti-CD8 (PerCP) and co-staining with anti-CD44 (PE) (BD Biosciences). 4-1BB was visualized with biotin-labeled anti-4-1BB (Biolegend) followed by FITC-labeled streptavidin (Molecular Probes). Intracellular staining for cytokine production in T cells was performed as previously described (Salek-Ardakani et al., 2008), with some modifications. Briefly, after lysing RBC, splenocytes from infected mice were resuspended in RPMI-1640 medium (Gibco) supplemented with 10% FCS (Omega Scientific), 1% L-glutamine (Invitrogen), 100 μg/ml streptomycin, 100 U/ml penicillin and 50 μM 2-mercaptoethanol (Sigma). 1–2 × 10^6^ cells were plated in round-bottomed 96-well microtiter plates in 200 μl with medium or the indicated VACV peptides at 1 μg/ml for 1 h at 37°C. GolgiPlug (BD Biosciences) was then added to the cultures according to the manufacture’s instructions and the incubation continued for 6–9 h. Cells were stained with anti-CD8 (PerCP) and CD62L (PE), followed by fixation with cytofix/cytoperm (BD Biosciences) for 20 min at 4°C. Fixed cells were subjected to intracellular cytokine staining in BD Perm/Wash buffer for 30 min at 4°C. Anti-TNF (FITC) and IFN-γ (APC) were obtained from e-Bioscience and used at a 1:100 dilution. Samples were analyzed for their proportion of cytoplasmic cytokines after gating on CD8^+^CD62L^low^ T cells by FACSCalibur^TM^ flow cytometer using CellQuest (BD Biosciences) and FlowJo software (Tree Star, san Carlos, CA).

### STATISTICAL ANALYSIS

Unless otherwise indicated, data represent the means ± SEM. Student’s *t*-test was used to determine statistical significance versus the control group. *p < 0.05.

## RESULTS

**FIGURE 1:**
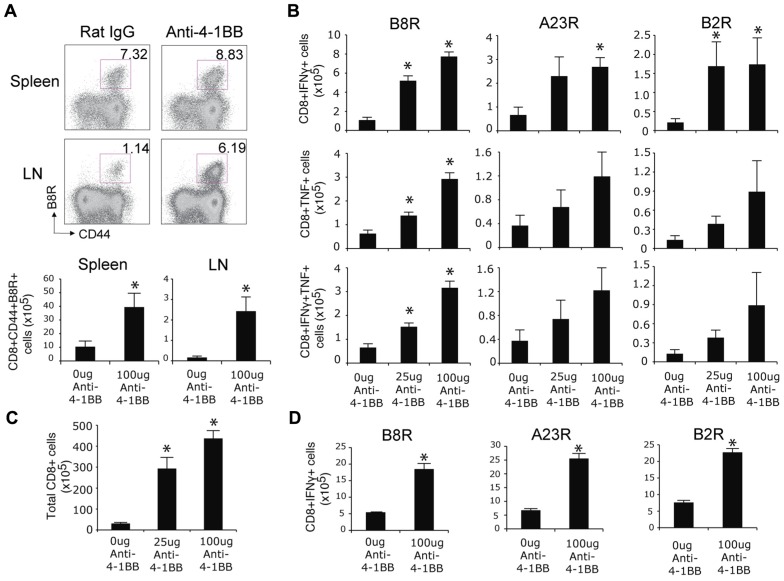
**Anti-4-1BB augments primary CD8 T cell responses to VACV-WR**. WT mice were infected i.p. with VACV-WR (2 × 10^4^ PFU/mouse) and treated with 0, 25, or 100 μg agonist anti-4-1BB or control antibody on day 1 post-infection. Seven days after infection, **(A)** Cells from spleen and peripheral lymph nodes (LN) were stained with anti-CD8, -CD44, and B8R-tetramer. *Top*: Representative dot plots of gated CD8^+^ cells. The numbers indicate the percentage of CD8^+^CD44^+^B8R-tetramer-positive cells. *Bottom*: Total number of CD8^+^CD44^+^B8R-tetramer-positive cells in the spleen and LN were calculated. **(B)** Splenocytes were stimulated with B8R, A23R, or B2R peptide, followed by staining for intracellular IFN-γ and TNF. Total numbers of CD8^+^IFN-γ^+^ (top), CD8^+^TNF^+^ (middle), and CD8^+^IFN-γ^+^TNF^+^ cells (bottom) were calculated. **(C)** Total splenic CD8 T cells numbers were determined. **(D)** Lung cells were stimulated with B8R, A23R, or B2R peptide, followed by staining for intracellular IFN-γ. Total numbers of CD8^+^IFN-γ^+^ cells were calculated. Data represent mean value ± SEM from *n* = 4 mice. Similar results were reproduced in two separate experiments. The Student’s *t*-test was used to determine statistical significance. *p < 0.05.

We previously found that endogenous 4-1BB/4-1BBL interactions played no apparent role in driving the CD8 T cell response following an infection with the VACV-WR (Zhao and Croft, 2012). However, we noted that 4-1BB was induced on VACV epitope-reactive CD8 T cells during the infection, apparent by day 2 and maintained for approximately 5 days (Zhao and Croft, 2012). This strong and prolonged expression of 4-1BB suggested that agonist reagents that targeted this molecule might still represent a means to boost CD8 T cell priming and be of potential use to augment immunity of relevance to smallpox infection or if VACV was used as a vaccine vehicle. To test this, mice were infected with VACV-WR and injected with an agonist antibody to 4-1BB, 1 day after infection to coincide with expression of the receptor. Even though VACV-WR induces a strong CD8 T cell response (Salek-Ardakani et al., 2011a), a single high dose of the agonist (100 μg) greatly enhanced (by four- to fivefold) the accumulation of primary effector CD8 T cells on day 7 in lymphoid organs, determined by flow analysis with a tetramer of the immunodominant peptide B8R (**Figure 1A**). Analysis of T cell reactivity further confirmed this after assessing the frequency of CD8 T cells capable of making IFN-γ alone, TNF alone, or both cytokines, following restimulation with B8R peptide *in vitro* (**Figure 1B**). The stimulatory effect of anti-4-1BB was not limited to the immunodominant CD8 T cell population as the frequency of several subdominant effector populations was similarly boosted four- to fivefold (**Figure 1B**, A23R, B2R). These data differ from prior studies of influenza where anti-4-1BB primarily augmented T cell responses to subdominant epitopes (Halstead et al., 2002). Increasing the dose of anti-4-1BB to 150 μg/mouse showed no further increase in the primary CD8 T cell response (not shown), and a lower dose of 25 μg/mouse still significantly augmented priming but generally to a proportionately lesser extent than 100 μg/mouse (**Figure 1B**). No difference was observed in the mean fluorescence intensity of staining for IFN-γ and TNF between the control and anti-4-1BB-treated groups (not shown) implying 4-1BB signaling primarily augmented expansion but not differentiation of the CD8 T cells. We also found that total CD8 T cells numbers increased following the antibody treatment (**Figure 1C**). These data correlate with prior data demonstrating the ability of 4-1BB to regulate division and survival of CD8 T cells (Takahashi et al., 1999; Cannons et al., 2001; Cooper et al., 2002; Lee et al., 2002, 2003). To determine if 4-1BB signaling led to accumulation of virus-reactive T cells in other organs, lungs were analyzed and consistent with the prior results anti-4-1BB also enhanced the number of CD8 effector T cells making IFN-γ in this organ (**Figure 1D**).

**FIGURE 2:**
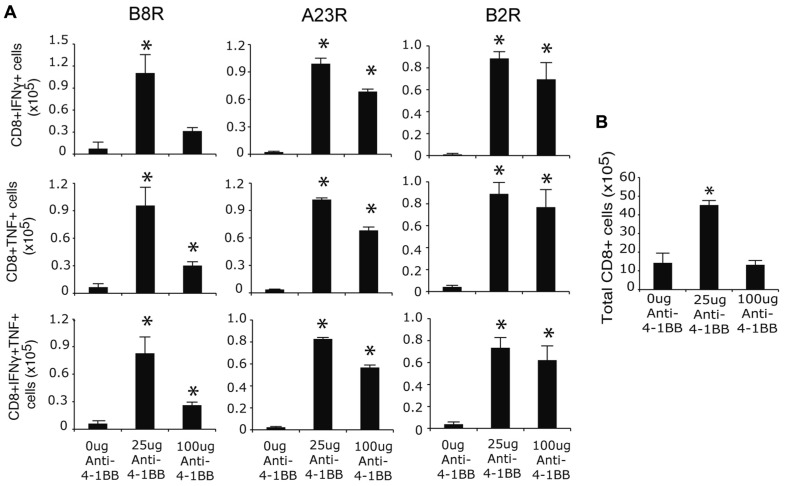
**Anti-4-1BB augments memory CD8 T cell responses to VACV-WR**. WT mice were infected i.p. with VACV-WR (2 × 10^4^ PFU/mouse) and treated with 0, 25 or 100 μg anti-4-1BB on day 1 post-infection. Thirty-five days after infection, **(A)** Splenocytes were stimulated with B8R, A23R, or B2R peptide, and stained for intracellular IFN-γ and TNF. Total numbers of CD8^+^IFN-γ^+^ (top), CD8^+^TNF^+^ (middle), and CD8^+^IFN-γ^+^TNF^+^ cells (bottom) were calculated. **(B)** Total splenic CD8 T cell numbers were calculated. Data represent mean value ± SEM from *n* = 4 mice. Similar results were reproduced in two separate experiments. Statistical significance. *p < 0.05.

To assess whether targeting 4-1BB promoted greater development of CD8 T cell memory, responses were assessed on day 35 when stable populations arise after VACV infection (Salek-Ardakani et al., 2008, 2011a). The frequency of memory CD8 T cells was again strongly boosted in mice injected with anti-4-1BB regardless of epitope-reactivity, and including polyfunctional T cells making both IFN-γ and TNF, as well as those making only IFN-γ or TNF (**Figure 2A**). In this case, the lower dose of anti-4-1BB was overall more effective in that fewer memory CD8 T cells were induced in mice treated with 100 μg of antibody, particularly within the B8R-reactive population. Similarly, total CD8 T cells numbers were increased following treatment with the lower dose of antibody (**Figure 2B**). Thus, targeting 4-1BB allows the more effective development of high frequencies of VACV-reactive effector and memory CD8 T cells during an infection with VACV-WR. Prior studies of anti-4-1BB in naïve mice demonstrated that a high dose (200 μg) resulted in a number of adverse events, including splenomegaly, lymphadenopathy, and hepatomegaly (Niu et al., 2007). In our experiments, we did observe splenomegaly in excess of that normally induced by the virus (approximately twofold more total spleen cells) at day 7, however, this was not seen at day 35 (not shown). The mice also showed no visible signs of adverse events based on daily monitoring during the studies (e.g., no weight loss, ruffled hair, lack of movement) and groups of mice that were not sacrificed for analyses survived over 18 months following anti-4-1BB treatment comparable to control antibody-treated groups.

**FIGURE 3:**
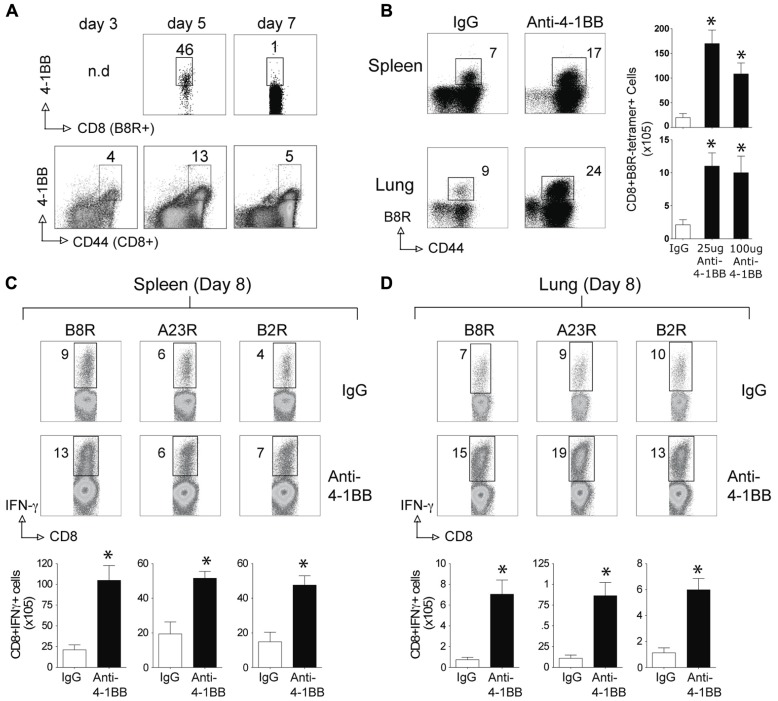
**Anti-4-1BB enhances the frequency of primary effector CD8 T cells after vaccination with VACV-Lister**. Mice were infected i.p. with 2.5 × 10^5^ PFU/mouse of VACV-Lister. One day later, mice were treated with 25 μg **(A–D)** or 100 μg **(B)** control rat IgG or anti-4-1BB and responses assessed on day 8. **(A)** Gated CD8^+^CD44^+^ cells and B8R-tetramer-positive CD8^+^ cells in spleens were stained for 4-1BB expression on days 3, 5, and 7. **(B)** Spleen (upper panel) and lung (lower panel) cells were stained with CD8, CD44, and B8R tetramer (left). Total numbers of CD8^+^ B8R-tetramer-positive cells were calculated in spleen (right). **(C,D)** CD8 T cell functionality in the spleen **(C)** and lung **(D)** was assessed by intracellular IFN-γ staining after stimulation with the indicated VACV peptides. Top: Representative plots of cytokine staining, gating on CD8^+^ T cells. Bottom: Total numbers of CD8^+^ IFN-γ^+^ cells per tissue. The results are means and SEM (*n* = 4 mice/group) from one experiment. Similar results were obtained in two separate experiments. Statistical significance. *p < 0.05.

We next tested whether stimulation of 4-1BB would similarly promote enhanced CD8 T cell responses to an attenuated VACV. VACV-Lister is significantly impaired in its ability to replicate compared to VACV-WR (Salek-Ardakani et al., 2011a), at least in part due to differential expression of genes or gene products that block innate immunity (Kettle et al., 1995; Alcami et al., 2000). Furthermore, and in contrast to VACV-WR, VACV-Lister given systemically, as in the protocols used here, cannot induce sufficient numbers of memory CD8 T cells to protect against a subsequent, normally lethal, infection with VACV-WR (Salek-Ardakani et al., 2011a). During infection with VACV-Lister we observed 4-1BB to be expressed on a large subset of B8R-reactive CD8 T cells at day 5 but it was down-regulated by day 7 (**Figure 3A**), in contrast to our prior observation with VACV-WR where ~30% of B8R-reactive T cells still expressed 4-1BB on day 7 (Zhao and Croft, 2012). We could not assess expression on B8R-tetramer-positive CD8 cells at an earlier time due to the low number induced by VACV-Lister. However, 4-1BB was up-regulated on a proportion of all effector/memory phenotype CD8 T cells (CD44-high-CD62L-low) as early as day 3, peaked at day 5, and was substantially reduced by day 7. Because 4-1BB is not constitutively expressed, many of these cells were likely reactive with other virus epitopes. Collectively this suggests that 4-1BB was only transiently expressed in response to Lister but was available as a target for the agonist antibody. Anti-4-1BB was again injected 1 day after VACV-Lister infection and primary effector CD8 T cells quantitated at the peak of the response on day 8. As with the response to VACV-WR, targeting 4-1BB resulted in a greater expansion of the B8R-reactive CD8 T cell population in the lymphoid organs as measured by tetramer analysis and intracellular cytokine staining, and also promoted enhanced accumulation of other epitope-specific populations (A23R, B2R) assessed by intracellular cytokine staining (**Figures 3B–D**). This was not restricted to the lymphoid organs as significantly greater numbers of VACV-specific CD8 T cells were visualized in the lungs. The effect of anti-4-1BB with VACV-Lister was generally similar to that observed with VACV-WR infection (three- to sixfold enhancement) and again primarily at the level of clonal expansion as production of cytokines (based on MFI) was largely comparable between the control and agonist antibody groups (**Figures 3C,D**, IFN-γ shown, TNF not shown). In this case, the dose of the antibody did not appreciably alter the extent to which expansion was affected in that a similar response was seen with 25 μg compared to 100 μg (**Figure 3B** and not shown).

**FIGURE 4:**
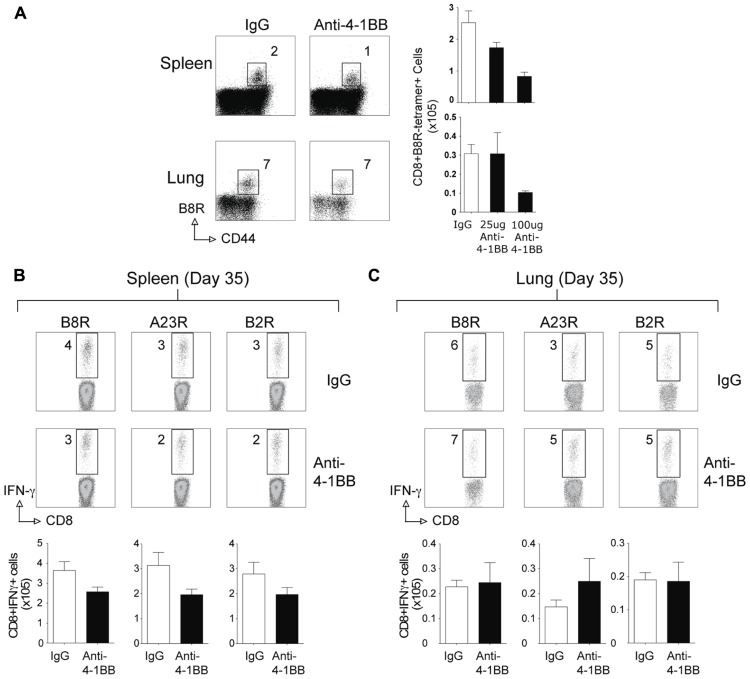
**Anti-4-1BB treatment does not enhance CD8 T cell memory to VACV-Lister**. Mice were infected i.p. with 2.5 × 10^5^ PFU/mouse of VACV-Lister. One day later, mice were treated with 25 μg **(A–C)** or 100 μg **(A)** of control rat IgG or anti-4-1BB and responses assessed after 35 days. **(A)** Spleen (upper panel) and lung (lower panel) cells were stained with CD8, CD44, and B8R tetramer (left). Total numbers of CD8^+^ B8R-tetramer-positive cells in spleens (right). **(B,C)** CD8 T cell functionality in the spleen **(B)** and lung **(C)** was assessed by intracellular IFN-γ staining after stimulation with the indicated VACV peptides. Top: Representative plots of cytokine staining, gating on CD8^+^ T cells. Bottom: Total numbers of CD8^+^ IFN-γ^+^ cells per tissue. The results are means and SEM (*n* = 4 mice/group) from one experiment. Similar results were obtained in two separate experiments. Statistical significance. *p < 0.05.

Surprisingly, when the memory CD8 T cell response to VACV-Lister was assessed, we observed no positive effect of anti-4-1BB treatment. The number of VACV-specific CD8 T cells was not enhanced in VACV-Lister infected mice when analyzed after 35 days (**Figure 4**), in contrast to the results in VACV-WR infected mice (see **Figure 2**). This was regardless of location assayed, and dose of anti-4-1BB injected. Collectively, these data suggest that targeting 4-1BB may be a useful method for augmenting the accumulation of viral antigen-specific CD8 T cells during infection with a VACV or VACV vector, but that the level of attenuation of the virus might determine whether the boosted response is maintained over time.

**FIGURE 5:**
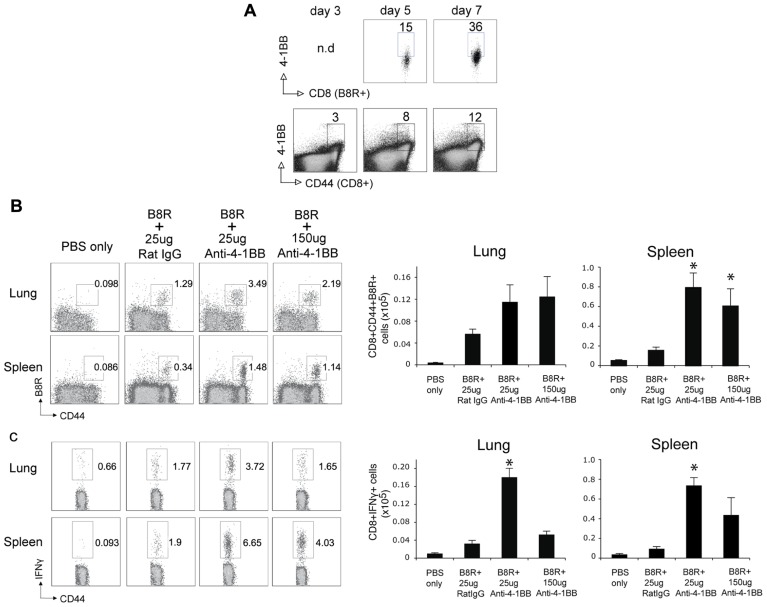
**Anti-4-1BB promotes the accumulation of memory CD8 T cells following VACV peptide immunization**. WT mice were immunized s.c. at the base of the tail with 2 μg of B8R peptide in IFA and 1 day later treated with 25 or 150 μg anti-4-1BB or control Rat IgG. Control groups received adjuvant but no peptide and antibody (PBS). **(A)** Gated CD8^+^CD44^+^ cells and B8R-tetramer-positive CD8^+^ cells in spleens were stained for 4-1BB on days 3, 5, and 7 post-immunization. **(B,C)** Three weeks after immunization: **(B)** cells from lung and spleen were stained with anti-CD8, -CD44, and B8R-tetramer. Left: Representative dot plots of gated CD8^+^ cells. The numbers indicate the percentage of CD8^+^CD44^+^B8R-tetramer-positive cells. Right: Total number of CD8^+^CD44^+^B8R-tetramer-positive cells in the lung and spleen; **(C)** cells from lung and spleen were stimulated with B8R peptide and stained for intracellular IFN-γ. Total numbers of CD8^+^IFN-γ^+^ cells were calculated. Data represent mean value ± SEM from *n* = 5 mice. Similar results were reproduced in two separate experiments. Statistical significance. *p < 0.05.

**FIGURE 6:**
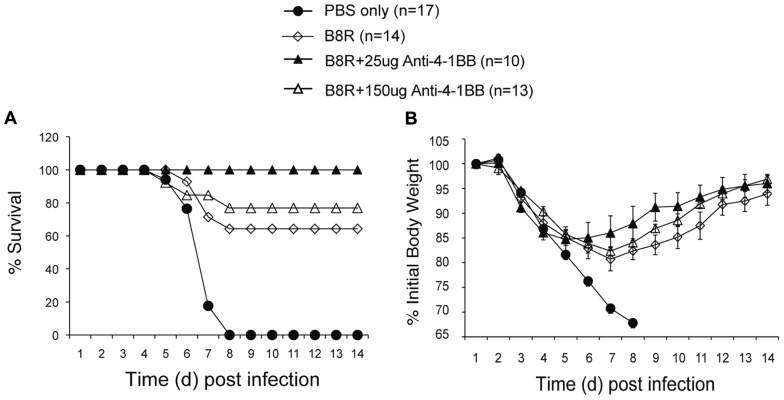
**Anti-4-1BB promotes protective CD8 T cells following vaccination with an immunodominant VACV peptide**. WT mice were immunized s.c. at the base of the tail with 2 μg of B8R peptide in IFA and injected with 25 or 150 μg anti-4-1BB antibody or rat IgG on day 1. Control groups received adjuvant but no peptide and antibody (PBS). Three weeks after vaccination, mice were infected intranasally with a lethal dose of VACV-WR [3.5 × 10^6^ PFU/mouse (300 × LD50)]. Animals were weighed daily and were euthanized if they maintained a weight loss of 25% or more for greater than 24 h. **(A)** Mean percent survival (*n* = 10–17/group). **(B)** Body mass presented as the mean % ± SEM of initial body weight. Data are pooled from three independent experiments with four to six mice/group.

Lastly, we determined whether targeting 4-1BB could promote protective CD8 T cell populations during vaccination with VACV antigens. Anti-4-1BB has previously been shown to boost memory generation with a non-viral peptide vaccination scheme (Myers et al., 2006). In our study, mice were immunized with the immunodominant VACV epitope, B8R, mixed in IFA, and 1 day later injected with 25 or 150 μg anti-4-1BB. 4-1BB was expressed on virus-reactive and total effector/memory CD8 T cells from day 3 to day 7. In contrast to VACV-Lister infection, although similar to VACV-WR infection (Zhao and Croft, 2012), 4-1BB expression was also found on ~35% of viral antigen-specific CD8 T cells 7 days after B8R immunization (**Figure 5A**). After 3 weeks, we found markedly increased accumulation of B8R-tetramer reactive CD8 T cells and increased frequencies of functional B8R-reactive cells assessed by staining for IFN-γ (**Figures 5A,B**). Again, this was regardless of location (spleen versus lung). The activity was most pronounced with a lower dose of anti-4-1BB, particularly evident in IFN-γ producing cells in the lung, but both doses tested strongly augmented the overall response. Next, we determined if these elevated frequencies of CD8 T cells were functionally significant. Mice immunized with B8R peptide and injected with anti-4-1BB were subsequently exposed to a normally lethal dose of VACV-WR given intranasally (**Figure 6**). We have previously shown that protection against this mucosal infection can be mediated exclusively by virus-specific CD8 T cells if their numbers are sufficiently elevated in the lungs (Salek-Ardakani et al., 2008, 2011b). Immunization with peptide alone provided some protection in that ~65% of mice survived, whereas treatment with anti-4-1BB enhanced the resistance to infection. This was more evident with low dose antibody treatment where 100% of mice survived (**Figure 6A**), correlating with the increased frequency of IFN-γ-producing CD8 T cells observed in the lungs above that present in mice injected with the high dose of antibody (**Figure 5**). No difference was observed in virus-induced pathology as assessed by weight loss of the mice over the initial 5 days of infection, however in mice surviving in either control or anti-4-1BB-treated groups, the lower dose of anti-4-1BB allowed the mice to recover their weight more quickly (**Figure 6B**). The latter suggested the low dose of anti-4-1BB antibody enhanced the quality of protection and resistance was primarily due to the expanded numbers of functional CD8 T cells.

**FIGURE 7:**
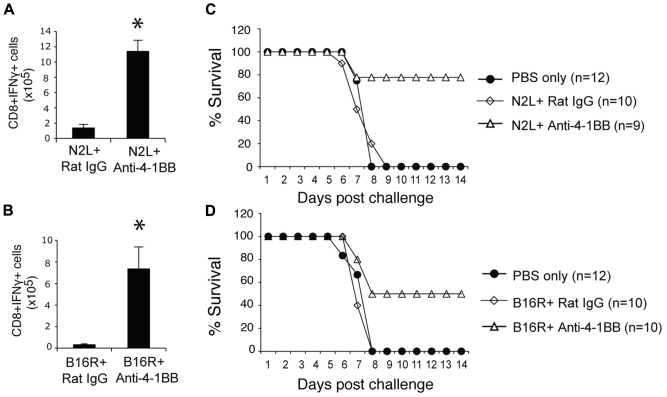
**Anti-4-1BB promotes protective CD8 T cells with vaccination with subdominant VACV peptides**. WT mice were immunized s.c. at the base of the tail with 10 μg of N2L **(A,C)** or B16R **(BD)** peptide in IFA and treated with 100 μg anti-4-1BB antibody or rat IgG as on day 1. **(A,B)** 7 days after vaccination, splenocytes from N2L peptide vaccinated animals were stimulated with N2L peptide, and splenocytes from B16R peptide vaccinated animals were stimulated with B16R peptide. Total numbers of CD8^+^IFN-γ^+^ cells were calculated. Data represent mean value ± SEM from *n* = 5 mice. **(C,D)** Three weeks after vaccination, mice were infected intranasally with a lethal dose of VACV-WR [3.5 × 10^6^ PFU/mouse (300 × LD50)]. Animals were weighed daily and were euthanized if they maintained a weight loss of 25% or more for greater than 24 h. Mean percent survival shown (*n* = 9-12/group). Data are pooled from two independent experiments with four to six mice/group. Statistical significance. *p < 0.05.

Lastly, we immunized with two subdominant VACV epitopes, N2L and B16R, again in IFA. Neither epitope alone was efficient in promoting the accumulation of epitope-reactive CD8 T cells, and this translated into no protection against a subsequent infection with VACV-WR (**Figure 7**). In contrast, anti-4-1BB given with IFA immunization strongly enhanced the numbers of IFN-γ-producing CD8 T cells that were generated, and engendered significant protection against infection with virulent virus. Thus, targeting 4-1BB may also be applicable to promoting anti-viral CD8 T cell populations induced by viral peptide vaccination as well as by live or attenuated viruses.

## DISCUSSION

Here, we show that treatment of mice with an agonist antibody to 4-1BB strongly promotes the expansion and accumulation of virus-specific primary effector CD8 T cells during infection with live virulent and attenuated VACV or with an immunization strategy with VACV peptides. This suggests that targeting 4-1BB may have application for boosting short-term CD8 T cell immunity when used in a vaccine setting with vaccinia. We also show that anti-4-1BB enhances the accumulation of VACV-specific memory CD8 T cells with live virulent virus and with virus peptides given in adjuvant, but not with attenuated VACV. This further suggests that targeting 4-1BB could be a useful strategy for long-term vaccination but that the type of vaccine may determine its usefulness and additional factors may need to be promoted to allow agonists to 4-1BB to enhance memory CD8 T cells responses with attenuated vaccinia viral vectors.

4-1BB has previously been shown to play a role in development of CD8 T cell responses to a number of viruses, although the requirement and stage of response for its activity has been reported to be variable. 4-1BBL^–/–^ mice were found to display reduced CD8 T cell responses to both influenza virus and LCMV Armstrong (DeBenedette et al., 1999; Tan et al., 1999; Bertram et al., 2002, 2004), with the defect seen in some cases during the primary response (Tan et al., 1999; Bertram et al., 2002), or late during memory stabilization after a normal acute response (DeBenedette et al., 1999), or primarily evident at the time of reactivation of the memory cells (Bertram et al., 2004; Hendriks et al., 2005). In other studies, 4-1BBL^–/–^ mice immunized with a lipidated version of an LCMV peptide had fewer primary and memory epitope-specific CD8 T cells and were impaired in their ability to resolve a subsequent LCMV infection (Tan et al., 2000). 4-1BBL^–/–^ mice also generated functionally impaired CD8 T cells during infection with latent mouse gammaherpesvirus-68 (MHV-68), but in this case their numbers were unaltered (Fuse et al., 2007), and 4-1BBL^–/–^ mice or wild-type mice injected with a neutralizing antibody to 4-1BBL generated lower late but not acute CD8 T cell responses to MCMV (Humphreys et al., 2010). These data combined have given good rationale for targeting 4-1BB in immunization or vaccination strategies for viruses as they directly demonstrate that 4-1BB is available to act as a stimulatory receptor. Proof of concept studies targeting 4-1BB with an agonist antibody, or 4-1BBL in a vector, consequently resulted in enhanced CD8 responses to influenza, LCMV, and HSV (Halstead et al., 2002; Kim et al., 2005; Zhang et al., 2007; Moraes et al., 2011; Vezys et al., 2011).

Our scenario was different in that we found no endogenous role for 4-1BB or 4-1BBL in driving a VACV-specific CD8 T cell response in studies with either gene-deficient mice or using a neutralizing anti-4-1BBL antibody (Zhao and Croft, 2012). However, we did find that 4-1BB was expressed on the VACV-reactive CD8 T cells shortly after infection with VACV-WR (Zhao and Croft, 2012) providing an interesting test of whether targeting this molecule would be efficacious with VACV immunization. The assumption by most in the field is that the requirement for 4-1BB to augment CD8 T cell expansion or activity is direct, based on *in vitro* data showing 4-1BB can induce signals in CD8 T cells that promote expression of cell cycle proteins and anti-apoptotic proteins (Cannons et al., 2001; Cooper et al., 2002; Lee et al., 2002, 2003; Nam et al., 2005). 4-1BB can be expressed on a number of cell types other than CD8 T cells, including activated CD4 T cells, NK cells, and NKT cells. However, expression of 4-1BB on the responding viral epitope-specific CD8 T cells is likely crucial for its activities in promoting CD8 T cell priming. We showed this in several non-viral models where TCR transgenic CD8 T cells lacking 4-1BB were tracked during responses driven by anti-4-1BB and failed to expand similar to wild-type T cells (Myers et al., 2006; Lee et al., 2007). This does not rule out effects of 4-1BB signaling on other cells but imply that expression of 4-1BB on the responding CD8 T cells will be critical to any vaccine protocol that incorporates a stimulant of this molecule.

Our data showed that anti-4-1BB effectively boosted primary effector CD8 T cell responses regardless of strain of virus or peptide immunization. These data extend other data with anti-4-1BB, or 4-1BBL inserted into a vector, that similarly showed enhanced initial priming of CD8 T cells to an HCV epitope expressed in adenovirus (Arribillaga et al., 2005) and to a DNA construct encoding HIV Gag (Ganguly et al., 2010). In all cases, targeting 4-1BB was effective in promoting either greater numbers of viral epitope-reactive CD8 T cells or CD8 T cells that had greater CTL activity. Interestingly, another study found that 4-1BBL inserted into a recombinant fowlpox vector expressing HIV Gag did not have any effect on augmenting a primary CD8 T cell response, but when it was used to boost a response at 2 weeks, 4-1BBL expression significantly augmented the subsequent accumulation of HIV-reactive CD8 T cells (Harrison et al., 2006). Why the latter study found no activity in the initial priming of CD8 T cells is unclear, but it is possible this was related to a lack of 4-1BB expression on the naïve CD8 T cells responding to the fowlpox vector, although no studies addressed this point.

Expression of 4-1BB may also be of relevance to our observations on memory generation. Although primary CD8 responses were boosted regardless of VACV virus or peptide immunization, we found the surprising result that anti-4-1BB only promoted enhanced memory with VACV-WR and peptide/IFA immunization but not with VACV-Lister. Although the explanation for this is not known at present, we think there are two possibilities. The first is that 4-1BB may simply not have been expressed on viral epitope-reactive CD8 T cells for a sufficient length of time during immunization with VACV-Lister. Whereas 4-1BB expression was observed on ~30% of viral antigen-specific cells over 7 days with VACV-WR infection or IFA/peptide immunization, few CD8 T cells expressed 4-1BB at this time with VACV-Lister. In contrast to VACV-WR, which persists for a long time, VACV-Lister is completely cleared from the ovaries between days 5 and 7 and the spleen at day 3 (Salek-Ardakani et al., 2008, 2011a). Thus, antigen persistence may result in prolonged expression of 4-1BB. Transient 4-1BB expression likely translated to a brief period of signaling from anti-4-1BB to augment clonal division in the effector cells, but the necessary pro-survival signals may not have been induced that result from extended 4-1BB signaling and that might be needed to allow greater accumulation of memory cells. Our findings are reminiscent of a prior study (Lin et al., 2009) that showed that 4-1BB expression on CD8 T cells was prolonged with respiratory tract infection of a virulent influenza virus compared to a milder influenza virus. This also correlated with a requirement for 4-1BB in the T cell response to the former, whereas 4-1BB was dispensable for the response to the less virulent strain.**Similarly, anti-4-1BB was previously shown to enhance primary T cell responses to influenza virus delivered by the non-productive i.p. route of infection, in which there is minimal viral replication, but had little effect on the accumulation of T cells at later times (Bertram et al., 2004). An alternative possibility is that attenuated/less virulent viruses may not induce additional co-signals that are needed to synergize with 4-1BB signals to augment T cell memory. This notion is based on our previous studies in non-viral systems where only peptide was used for immunization, which results in little/no generation of memory CD8 T cells. In one case, anti-4-1BB combined with a TLR3 ligand (poly IC) resulted in accumulation of memory CD8 T cells, whereas the agonist alone or the TLR3 ligand alone had no effect (Myers et al., 2006). In another study, anti-4-1BB combined with a second agonist to a different TNFR superfamily molecule, OX40, also allowed the survival of polyfunctional CD8 T cells with peptide immunization, whereas neither agonist alone was very effective (Lee et al., 2007). Thus, anti-4-1BB might provide some of the necessary survival signals that allow memory generation or persistence, but additional synergistic signals from either cytokines like IFN-α or IL-12 that might be induced by TLR ligands, or from other TNFR family molecules, may be a prerequisite for this activity. Therefore, sustained viral antigen presentation may regulate prolonged expression of 4-1BB on responding T cells, and a long-lasting inflammatory environment may provide additional co-signals that are needed for anti-4-1BB to promote memory. The exact nature of any required co-signals might vary, but they are likely to be provided by virulent viruses like VACV-WR and certain influenza strains, but not by attenuated viruses such as VACV-Lister, explaining the dichotomous results on memory generation. IFA immunization with peptide, as we performed in the current study, most likely also allows sustained antigen presentation and provides an inflammatory environment that fulfills both of these requirements.

In summary, our studies demonstrate that targeting molecules like 4-1BB may provide sufficient adjuvant-type activities to allow vaccination with attenuated poxvirus vectors or viral antigens to promote effective short-term CD8 T cell immunity. However, our results question whether targeting molecules like 4-1BB in isolation will be sufficient for allowing long-term CD8 T cell responses to attenuated viruses or viral vectors, and warrant future studies of combination targeting that could engender the appropriate signals to also provide effective CD8 T cell memory.

## Conflict of Interest Statement

The authors declare that the research was conducted in the absence of any commercial or financial relationships that could be construed as a potential conflict of interest.
